# Comparing Biological Motion Perception in Two Distinct Human Societies

**DOI:** 10.1371/journal.pone.0028391

**Published:** 2011-12-14

**Authors:** Pierre Pica, Stuart Jackson, Randolph Blake, Nikolaus F. Troje

**Affiliations:** 1 Unité Mixte de Recherche 7023, Centre National de la Recherche Scientifique, Saint-Denis, France; 2 Laboratoire Structure Formelle du Langage, Université Paris 8, Saint-Denis, France; 3 Department of Psychology, Vanderbilt University, Nashville, Tennessee, United States of America; 4 Brain and Cognitive Sciences, Seoul National University, Seoul, Korea; 5 Department of Psychology, Queen's University, Kingston, Canada; Northwestern University, United States of America

## Abstract

Cross cultural studies have played a pivotal role in elucidating the extent to which behavioral and mental characteristics depend on specific environmental influences. Surprisingly, little field research has been carried out on a fundamentally important perceptual ability, namely the perception of biological motion. In this report, we present details of studies carried out with the help of volunteers from the Mundurucu indigene, a group of people native to Amazonian territories in Brazil. We employed standard biological motion perception tasks inspired by over 30 years of laboratory research, in which observers attempt to decipher the walking direction of point-light (PL) humans and animals. Do our effortless skills at perceiving biological activity from PL animations, as revealed in laboratory settings, generalize to people who have never before seen representational depictions of human and animal activity? The results of our studies provide a clear answer to this important, previously unanswered question. Mundurucu observers readily perceived the coherent, global shape depicted in PL walkers, and experienced the classic inversion effects that are typically found when such stimuli are turned upside down. In addition, their performance was in accord with important recent findings in the literature, in the abundant ease with which they extracted direction information from local motion invariants alone. We conclude that the effortless, veridical perception of PL biological motion is a spontaneous and universal perceptual ability, occurring both inside and outside traditional laboratory environments.

## Introduction

Carefully crafted psychological tasks can allow us to peek into the minds of other people, figuratively speaking, drawing inferences about underlying cognitive and perceptual processes. When applied to people living in cultures very different from ours, such tasks can answer fundamental questions about the nature of mental representations and the dependence of those representations on biological and cultural influences. This latter line of investigation has a long and rich history in experimental psychology [1], and it continues to bring insights [2] and surprises [3]. In this report, we present results from specially constructed tests of visual perception administered to an indigene group, the Mundurucu. These individuals live in a remote region of the Amazon shielded from cultural artifacts ubiquitous in so many other cultures around the world, including television and video animation. Prior behavioral research with Mundurucu volunteers has yielded important findings, related particularly to the development of the number sense and geometrical reasoning [2], [4], [5]. For example, in a series of elegant experiments that capitalized on the small number lexicon that exists in the Mundurucu language, researchers have found evidence providing strong support for important distinctions between exact and approximate arithmetic [4]. In the current study, we examined the perception of biological motion amongst Mundurucu volunteers, in an attempt to gather the first true set of field data on this important perceptual ability (for loosely related work, see [6]).

Our study employed point-light (PL) video technology [7] to create animations portraying walking animals and humans. With these unique animations, the changing positions of the limbs, trunk and head of the organism are defined by the shifting positions of small light spots strategically attached to different body parts (stimulus examples can be found at http://www.biomotionlab.ca/Demos/scrambled.html). Because individual frames of these PL sequences do not explicitly portray the body or how its parts are moving, the successful perception of biological activity must rely, at least in part, on the kinematics portrayed by the sequences. When viewing PL animations, most people can readily identify the activity [7], [8] and the species [9] and, in some circumstances, the actor's gender [10], [11], emotional state [12] or identity [13]. Although perception of biological motion has been widely studied in recent years [14], prior to our study it was unknown whether our effortless skills at perceiving biological activity from PL animations generalizes to people who have never before seen representational depictions of human and animal activity. Using well established experimental protocols inspired by over 30 years of laboratory experiments, we sought to address this issue by exploring perception of PL biological motion amongst Mundurucu volunteers. The results of these experiments, and identical experiments carried out with a group of American observers, are presented below.

## Materials and Methods

### Participant recruitment and ethics statement

Informed consent was obtained from all participants in our study, following procedures and ethical guidelines as detailed below.

**Figure 1:**
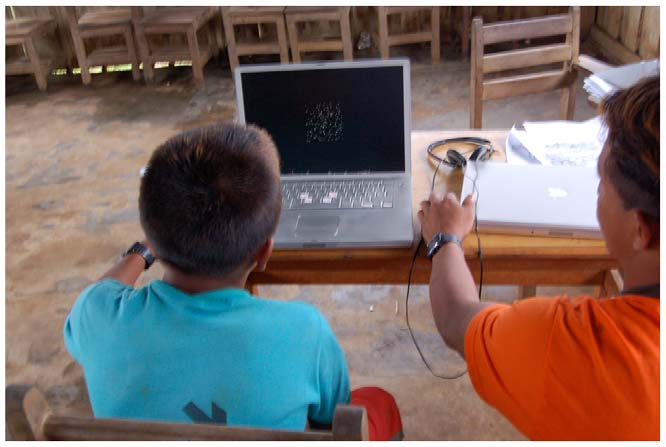
Experimental set-up. Mundurucu observers viewed stimuli that were presented on a solar-powered laptop computer. Most observers came from the isolated village of Muissu, located south of Jacareacanga along Rio Cururu, in the Para region of Amazonia. Photo taken in Muissu, copyright Pierre Pica/CNRS.

The native Brazilian group comprised 36 Mundurucu volunteers (mean age 22.1, range 8–60 years, 28 males); most were recruited at Muissu, an isolated village upstream from the Cururu mission in the Mundurucu main territory, which is located in the state of Para, Brazil (Figure 1). The Amazonian data was collected by author PP, with the ethical approval of the Fundação do Indio (FUNAI) and the Centre National de la Research Scientifique (CNRS), Paris, and in accordance with the standards described in the Declaration of Helsinki. FUNAI is the Brazilian government body in charge of protecting the rights of indigenous groups and controlling outsider access to the protected territories. After an invitation to take part in the study, informed verbal consent was obtained from all Mundurucu volunteers who participated, a process which was documented by the commencement of the computer program running the experiment. Informed verbal consent was also obtained from parents/guardians where appropriate (for study participants under 18 years of age). Verbal consent was deemed preferable given the typically low levels of formal education/reading ability amongst Mundurucu volunteers. This decision was made with the agreement of FUNAI, and is in accordance with the principles of the American Anthropological Association relating to the specific form of informed consent (see AAA Ethics Task Force).

In separate experiments, data was collected (by SJ) from 36 American volunteers (mean age 24, range 18–46 years, 21 males) tested at the Department of Psychology, Vanderbilt University. Informed written consent was obtained from all American volunteers, and testing at Vanderbilt was carried out in accordance with the guidelines of the local Institutional Review Board. All American volunteers were born in the USA, and were drawn mostly from the volunteer pool or student/staff body of Vanderbilt University.

### Stimuli

Biological motion stimuli were based on three different PL animation sequences presented at natural walking speed: a walking cat, a walking pigeon and a walking human. In the remainder of the paper, the term ‘walkers’ refers to the collection of all three animate motion types. All three sequences depicted a sagittal view of stationary, treadmill walking (meaning there was no net lateral motion). All other stimuli were derived from these veridical, upright PL displays by inverting the display and/or scrambling it spatially and temporally. The number of dots specifying the motion of the feet was different in the three different creatures. In the human figure, only the two ankle markers were used. In the pigeon, four markers were considered part of the feet (the two front and the two back toes), and in the cat, six markers (the ankle and toe on each front leg and one toe marker on each hind leg) were considered to belong to the feet. Further details on stimulus construction can be found in Text S1. Stimulus examples can be found at http://www.biomotionlab.ca/Demos/scrambled.html

### Experimental design

We collected data in two experiments. The task in both was a standard walker direction discrimination judgment, in which observers deciphered the facing direction of PL stimuli presented in noise (i.e., leftward vs. rightward). The experiments differed primarily in the manipulations made to the stimuli (e.g., inversion and/or scrambling), in the number of test trials, and in the stimulus presentation duration. The motivation for these differences is explained below.

In Experiment 1, PL displays could appear either upright or inverted, and either coherent or scrambled. Inversion was implemented by mirror flipping the walker about a horizontal axis. Scrambling was achieved by offsetting the location of each individual dot by a random amount within the area occupied by the unscrambled stimulus, and randomizing the phase relations between the different dots. Thus there were four main trial categories: upright coherent, inverted coherent, upright scrambled, inverted scrambled. Observers completed 96 trials in total, with equal numbers of trials per condition presented in random order. Conditions were equated in terms of the number of trials per walker facing direction (left/right) and animate motion type (human, pigeon, cat). Stimuli were embedded in a mask (9.3×9.3 degrees) of 100 randomly positioned stationary dots with a limited lifetime of 170 ms, and remained on screen until the observer responded with a left or right button press (2AFC). Trials were interleaved with a 1.2 s intertrial interval following the response. The set-up in Experiment 1 was thus a direct replication of experiments in [15]. We chose this design for our first experiment for several reasons. From an experimental point out view, we could obtain an immediate impression of whether Mundurucu volunteers perceived upright, coherent PL walkers veridically, as well as testing them on the more difficult task of extracting direction information from scrambled biological motion. From a practical point of view, the experiment is brief enough to be successfully administered under challenging conditions (e.g., via a solar-powered laptop), taking ∼10 mins to run.

In Experiment 2, we reduced drastically the stimulus presentation duration in an attempt to more rigorously study and compare observer sensitivity to local motion invariants in biological motion (i.e., observers' sensitivity to direction from upright scrambled walkers, see [15]). Thus, only upright stimuli were presented, both coherent and scrambled, and again embedded in a mask of 100 flickering noise dots (lifetime 125 ms). Observers completed 192 trials, during which the stimulus appeared for only 1 s on each trial. After this brief stimulus display, two arrows (left/right facing) appeared on screen, prompting the participant to respond. After the observer responded, a 1.2 s intertrial interval preceded the next trial. Note that the coherent walker condition was included specifically to make the task more meaningful and rewarding for observers; the primary objective was to study observer performance on the upright scrambled walker conditions under threshold conditions. We chose the trial settings above as they have been found in previous work to give a direction discrimination performance of approximately 75–80% with upright scrambled walkers (see Fig. 3B in [16], second data point for scrambled condition). In addition, by increasing the number of trials while limiting the task to only upright conditions, we could also compare observer performance for the various animate motion types more thoroughly (with 32 upright scrambled trials per category compared to 8 trials in Experiment 1).

### Testing procedure

Participants were told that they would see a number of different people/animals in motion and that they would have to decide in which way the animate creature was facing (using native phrases chosen appropriately for testing with Mundurucu volunteers). For each experiment, the instructions were followed by a practice block consisting of 18 trials. The first 6 practice trials consisted of unmasked walkers (each of the three animals shown in left/right orientation), so that participants could achieve a clear understanding of the left/right direction discrimination task without difficulty. For the Amazonia-based experiments, stimuli were presented on a portable, solar-powered Apple laptop (Powerbook G4, 15.2”). During testing, participants were seated in front of the laptop, with no set restrictions on viewing distance. Tests were administered in a well lit room. Testing with American participants was carried out using a Mac Pro/LCD display set-up (a similar laptop was not available). While the LCD display was initially set a fixed distance from the table edge where participants sat, participants were encouraged to modify the screen's distance or tilt angle so as to achieve the most comfortable viewing set-up (thus viewing distance was also not specifically controlled). Screen size and resolution were chosen to provide a close match to the laptop in terms of stimulus size, resolution and aspect ratio.

### Additional test and analysis

Before performing this study we anticipated the possibility that the Mundurucu might exhibit relatively poor performance on the biological motion task, and if that indeed was the result, we wanted to know whether that outcome might be attributable to non-perceptual factors such as motivation or attention. Thus, we included an additional task in our test battery, a task that did not involve biological motion. This task required participants to identify a partially fragmented, octagon-shaped geometric form that was embedded within noise contours. This task is quite challenging and, if performed successfully, it would provide independent evidence of an individual's ability to maintain focused attention and motivation during a series of discrete trials in which the individual tried to detect a masked figure within masking noise. As things turned out, motivation and attention were not issues in our main experiment, so for sake of brevity and focus of the main paper, we provide information and results on this additional task in Text S1). Further details on our main analysis (e.g., some minor data exclusions) and on PL stimulus construction are also documented there.

## Results

### Experiment 1

**Figure 2:**
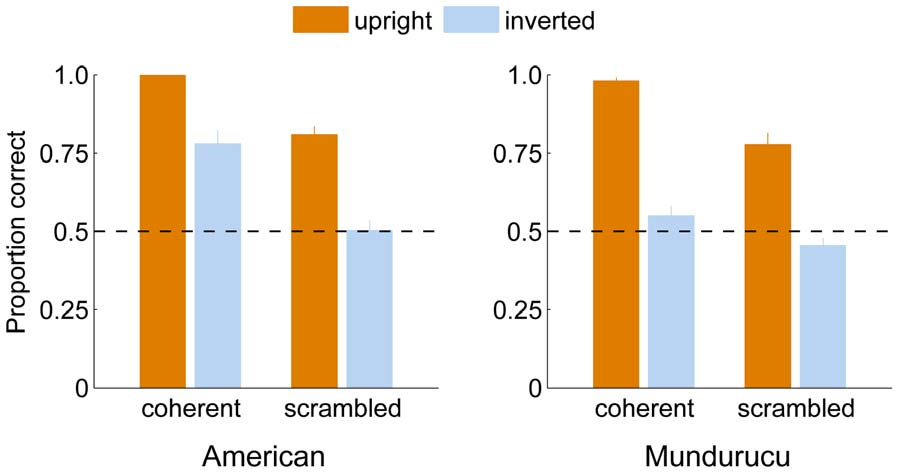
Direction discrimination accuracy in Experiment 1. Barplots depict the mean direction discrimination scores (*n* = 18 each group) for each of the four main conditions, presented separately for American and Mundurucu observers. Both global and local inversion effects are apparent for each group, with direction discrimination performance on upright trials significantly greater than for inverted trials. Notably, direction is readily retrieved from upright scrambled walker trials. Error bars represent +1 SE.

Figure 2 summarizes the proportion correct responses from Experiment 1. A 2 (group)×2 (coherence)×2 (inversion)×3 (animate motion type) repeated measures analysis of variance (ANOVA), with group as between-subjects factor, revealed several main effects and interactions. The main effects for walker coherence and orientation were significant (coherence: F(1, 34) = 168.1, p<10^−12^; orientation: F(1, 34) = 153.3, p<10^−12^). On average, inversion reduced the accuracy of observers from a proportion correct of 0.99 to 0.66 in coherent walker conditions, and from 0.79 to 0.48 in scrambled walker conditions. The group main effect was also significant (F(1, 34) = 10.72, p<0.005; mean proportion correct: Mundurucu 0.69, American 0.77), as were several group-based interactions. Inversion appeared to have a greater effect in reducing Mundurucu performance on coherent walker trials, as indicated by the significant group×coherence×orientation interaction (F(1, 34) = 8.37, p<0.01; upright/inverted coherent means: Mundurucu 0.98/0.55, American 1.0/0.78). There was no main effect of animate motion type on performance (F(2, 68) = 0.54, p = 0.58), although a number of animate motion-based interactions were significant, including the three-way interaction of coherence×orientation×animate motion (F(2, 68) = 6.97, p<0.005). This effect appeared to be driven by considerably lower accuracies with upright scrambled human walkers (0.68) compared to upright scrambled trials for either of the other two animal types (cat: 0.82, pigeon: 0.88).

To summarize, the results of our first experiment illustrate several things. First, Mundurucu accuracy was close to 100% for upright coherent walker trials, using stimuli which were drawn from three different animate motion types. In other words, Mundurucu observers readily perceived the direction of upright, coherent PL walkers on their first exposure to such stimuli. In addition to the classic inversion effect for coherent walker stimuli, Mundurucu observers also experienced an inversion effect for scrambled motion stimuli. Notably, however, direction discrimination performance with upright scrambled walker displays remained well above chance, replicating recent findings [15] and supporting the notion of an orientation-specific visual filter for detecting local animate motion signals (see also [17]). These findings, as well as within condition differences due to group/animate motion, are elaborated on more fully in the Discussion.

### Experiment 2

**Figure 3:**
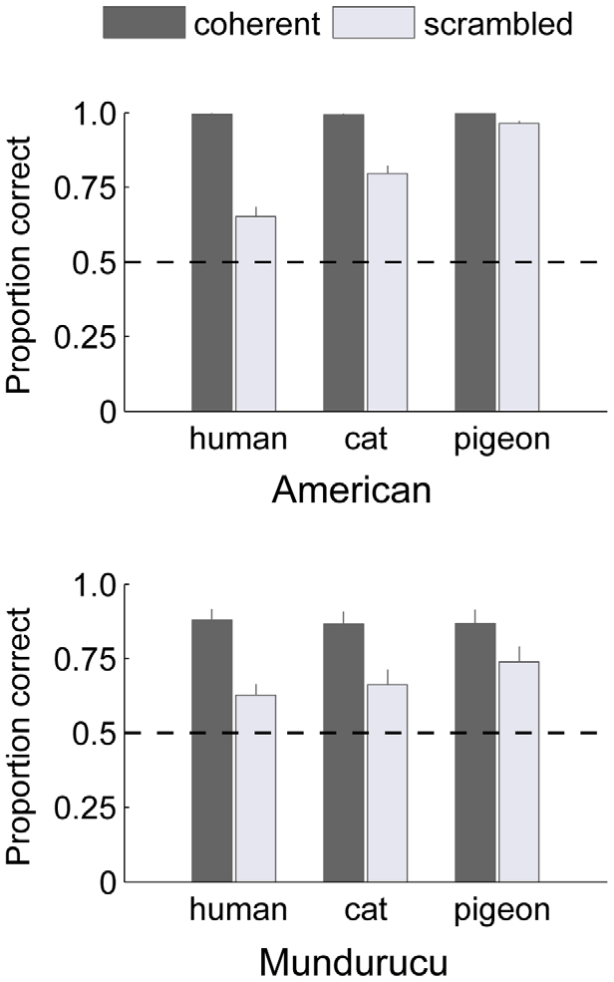
Direction discrimination accuracy in Experiment 2. The upper barplot depicts the mean direction discrimination scores for American observers (*n* = 18) in Experiment 2, presented separately for each animate motion type and coherence level (upright stimuli only). The lower barplot depicts the same data for Mundurucu observers (*n* = 18). Error bars represent +1 SE.

Figure 3 summarizes the proportion of correct responses from Experiment 2. A repeated measures ANOVA revealed several main effects and interactions. There was a significant main effect for coherence (F(1, 34) = 166.76, p<10^−12^). A significant main effect of group was also observed, given that on average, Mundurucu observers scored lower than American observers on this task (F(1, 34) = 9.19, p<0.005). The main effect for animate motion type was significant (F(2, 68) = 44.7, p<10^−12^), as were each of the animate motion interactions, including the interaction with coherence level (F(2, 68) = 53.04, p<10^−12^). A similar trend is apparent for both Mundurucu and American observers when it comes to the category of animate motion observed, although this trend was noticeably less pronounced in the Mundurucu data (Figure 3; group×coherence×animate motion interaction F(2, 68) = 9.67, p<0.0005). Specifically, in agreement with the findings from Experiment 1, both groups performed with mean direction discrimination accuracy for pigeon>accuracy for cat>accuracy for human scrambled motion trials (Mundurucu: 0.74>0.66>0.63; American: 0.96>0.8>0.65). Post-hoc tests confirmed that within individual observer groups, accuracy for pigeon motion trials was consistently better than for either of the other two animate motion types. Accuracy for cat motion trials, however, did not differ significantly from accuracy for human motion trials in the Mundurucu group (paired t-tests, Bonferroni corrected, Mundurucu: pigeon vs human, t(17) = 3.43, p<0.0032; pigeon vs cat, t(17) = 3.1, p = 0.0065; cat vs human, t(17) = 1.28, p = 0.22; American: all three tests p<10^−4^). Note that we believe this consistent trend is related not to the ‘species’ of stimulus per se, but to certain parameters of motion that varied within our particular stimuli (e.g., degree of vertical acceleration), a point we spell out more clearly in the Discussion.

To summarize, accuracy was again very high for upright coherent walker conditions. Performance fell for scrambled compared to coherent walker trials, yet remained well above chance level for all categories of scrambled stimuli and for both groups of observers. Notably, when the scrambled walker trials are broken down by animate motion type, findings from Experiment 2 appear to be in general agreement with the trend observed in Experiment 1.

## Discussion

Many laboratory investigations have confirmed that human observers are quite adept at detecting representational depictions of human activity presented in masked displays [18]–[20]. For example, human observers can effortlessly detect a PL walker embedded in scrambled walker noise [18]. More recently, it has been found that observers can perform above chance at discriminating walking direction from scrambled PL stimuli, stimuli that contain no obvious human or animal form [15]–[17], [21]. In fact, laboratory findings to date suggest that at least two complementary mechanisms underpin biological motion perception in humans – a rapidly-activated mechanism tuned to salient local motions in the image such as those caused by moving feet [15], [16], [22], and a global integration mechanism that recovers the articulated structure of the body and pieces together individual postural representations as they unfold over time [18], [23]. Until now, however, no study has extended these findings beyond traditional laboratory settings.

The results of our experiments consist of several important and novel findings. First, Mundurucu observers, individuals with no prior exposure to representational depictions of human or animal activity (i.e., PL animations), nevertheless appeared to have a very immediate and accurate sense of the animate motions depicted in our stimuli. In Experiment 1, direction discrimination performance with upright, coherent PL walkers was close to perfect, and observers experienced the same decremental inversion effects documented many times in laboratory based experiments [15], [17], [24]. While the inversion effect experienced by Mundurucu observers for coherent stimuli was more substantial than that experienced by our American observers, we note that chance performance on this condition is in line with prior findings from laboratory based experiments [15], [17]. Clearly the most illuminating result from our first experiment concerns the scrambled walker conditions: from stimuli that depict no animate form of any sort, Mundurucu observers performed well above chance at deciphering the underlying direction information. With this simple experiment we have, to the best of our knowledge, collected the first truly cross-cultural field data on PL biological motion perception.

One could argue that we already knew that experience with representational depictions of human and animal activity is not essential for effortless perception of biological motion - this is implied by the evidence showing that very young infants can distinguish upright from inverted PL animations of a walking hen [25]. Using a preferential looking paradigm, the researchers in that study found that at 2 days of age neonates already preferentially looked towards the biological motion stimulus (more so than towards an inverted version or a set of random dots). These earlier results, and our present results, are obviously relevant to the question of the ‘innateness’ of perceptual mechanisms dedicated to biological motion. As a scrambled biological motion condition was not included in the infant study, however, one cannot say conclusively whether the 2-day old infants were responding to upright local motions or to upright articulated shapes. We conclude from the neonate data that infants may already be sensitive early on to the orientation of local aspects of biological motion, but that they most likely do not care much about articulated shapes. Our study, in contrast, comes at the ‘innateness’ question from another angle – in two distinct human populations, one of which has no prior experience with representational depictions of animate motion, we find very similar sensitivities on local biological motion perception tasks using a range of different animate motion varieties. Together, the two strands of research converge on the idea that important and perhaps innate components of the machinery dedicated to biological motion consist of those mechanisms that are sensitive to the local invariants in biological motion.

How do our results extend the theoretical understanding of brain mechanisms dedicated to biological motion perception? When broken down by animate motion type, results from our experiments suggest that direction discrimination performance on upright scrambled walker conditions is specific to the type of animate motion viewed (although still well above chance for each of the categories presented). What is the source of this differential performance? Although one could envisage a scenario whereby heightened sensitivity to certain non-human animate motions is evolutionarily favorable, we do not believe that this is a truly plausible explanation for this effect. We did after all employ only one ‘standard’ exemplar for each of the three categories of animate motion, and besides, such an explanation would hardly lead to our observed pattern of results (i.e., with the direction of local motions from pigeon walking more easily discriminated than direction from four-legged cat walking).

Rather, it is likely that those properties of the local motion signal that are most information-heavy for performing life detection and direction discrimination (e.g., vertical acceleration of the feet) are available to greater or lesser degrees across the three animate motion types tested. Given that a constant noise density was used for each animate motion type in our experiments, these differences in vertical acceleration and displacement profile may have been differentially masked by the noise. In support of this interpretation, one previous study has found the three animate motion varieties used here to be differentially effective for discriminating direction [22]. In that study, stimuli containing only the foot motions were used, making a direct comparison with the current results inappropriate. Considered in this general light, however, we believe that our results support the view that perceptual mechanism(s) sensitive to local invariants in biological motion are tuned to a single parameter or well-defined subset of motion parameters (e.g., vertical acceleration of the feet). This might explain the consistent trend we found in both experiments for weaker performance with human compared to pigeon scrambled motion trials. This conclusion also naturally leads one to ask what the proper domain of specialization of these mechanisms is, and in line with the interpretation of earlier findings [15], [22], we suggest that these mechanisms are tuned broadly for “life detection” of animate creatures in general. That is, the phylogenetic development of these early visual detection mechanisms was presumably not driven by human-human interaction per se, but more generally by predator-prey interaction.

Either way, what is clear from our current results is that this sensitivity is remarkably similar across observers and distinct populations of observers. Mundurucu observers demonstrated effortless, veridical perception of PL biological motion, adding weight to the conjecture that this is a spontaneous and universal perceptual ability, occurring both inside and outside traditional laboratory environments.

## Supporting Information

Text S1 **This supplementary text provides information and results on an additional task that participants completed, as well as further details on our main analysis and on stimulus construction.**
(PDF)
