# Supplementary material for: Comparing Biological Motion Perception in Two Distinct Human Societies
Source: PLoS One. 2011 Dec 14;6(12):e28391. doi: 10.1371/journal.pone.0028391 (PMC3237441; doi:10.1371/journal.pone.0028391)
Supplement: Text S1 — This supplementary text provides information and results on an additional task that participants completed, as well as further details on our main analysis and on stimulus construction. (PDF) [file pone.0028391.s001.pdf]

Supplementary information

Pica et al. - Comparing biological motion perception in two distinct human societies

Analysis

Standard statistical procedures were used throughout (ANOVA, t-test). Data from equal numbers of Mundurucu and American observers (n = 36 total for each group) were analyzed, with unique sets of n = 18 observers taking part in each experiment. Prior to these analyses, some minor but unavoidable data exclusions were made in order to maintain the integrity of our participant groups in terms of: (i) the requirement for observers to have no previous experience with biomotion stimuli, and (ii) the requirement for observers to have been born and raised entirely in one strict geographically-defined location. Specifically, a number of Mundurucu observers completed the walker trials of Experiment 1 *having previously* taken part in Experiment 2. As these observers were no longer naive to biological motion tasks on completing the Experiment 1 trials, their data for Experiment 1 was not used in the analyses with the other eighteen observers. Data from one American observer was excluded from analysis on similar grounds. While running in Experiment 1, this observer noted having taken part previously in a study that employed biological motion stimuli. Data from two additional American observers was excluded when they revealed to the experimenter that, while being US citizens, they were born outside the country and had moved to the US as children.

Further details about PL stimulus construction

The human PL walker represented the average walker computed from a database consisting of motion-captured data from 50 women and 50 men. Details about the data acquisition and about the algorithm used to average the 100 walkers can be found elsewhere [1]. A set of 11 markers was used, which represented the head, one shoulder, one hip, the two elbows, two wrists, two knees, and two ankles. The second point-light sequence showed a walking pigeon. Data were recorded from a pigeon that was trained to walk back and forth between two feeders. The pigeon was supplied with 11 retroreflective markers. Both humans and pigeons were recorded by means of an optical motion capture system (Vicon, Oxford Metrics); constant translation was subtracted from the data, and a third-order Fourier series was fitted to the data to smooth them and loop them into a repeatable walking cycle. The third sequence showed a walking cat. The data are based on a high-speed (200 fps) video sequence showing a cat walking on a treadmill. Fourteen feature points were manually sampled from single frames. As with the other sequences,

data were approximated with a third-order Fourier series to obtain a generic walking cycle. Gait frequencies were 0.93 Hz for the human, 1.6 Hz for the pigeon, and 1.7 Hz for the cat. The display size on the screen was 2.3 x 5 degrees of visual angle for the human, 3.1 x 4.4 degrees for the pigeon, and 5.7 x 3.1 degrees for the cat.

Additional task

Prior to completing the walker task in Experiment 2, observers also completed a contrasting global form perception task. This task is a version of the "pathfinder" display devised by Field et al. [2] and used in earlier work by one of us, where a more detailed description can be found [3,4]. Briefly, on each of 100 test trials (two concurrently-run 50 trial staircases) the entire laptop monitor was filled with short lines whose orientations were randomly determined on each trial. The display was also divided into four equal-sized quadrants, clearly defined by thick black lines. On each trial, a group of six lines forming a quasi-circular figure (hexagonal) was presented at a randomly chosen location within one of the four quadrants, and observers' task was to detect this hidden target on each trial (by noting which quadrant it was in). Over trials the quasi-circular figure could appear in any of the four quadrants with equal probability. To gauge the sensitivity of our observers ($n = 19$ for each group), the clarity of the figure was manipulated by adding *jitter* to the orientation of the six linear elements forming the circular figure (with jitter defined as a range of angles over which individual lines belonging to the figure could deviate from the value specified by their position on the circle). Feedback on each trial was provided by highlighting the hidden pseudo-circular form in yellow following participant response. Formal testing was preceded by a short practice block consisting of 16 trials, which began with easy zero-jitter trials. Threshold values were calculated as the mean over the last 8 trials of a staircase procedure.

Detecting this simple static figure in a noisy background is a rather demanding task, and presumably relies upon early visual processing stages where lateral interactions between orientation-selective neurons are thought to support contour integration. Performance on this task, defined as the amount of angular jitter at which the observer could locate the pseudo-circular figure with approximately 75% accuracy, did not differ between groups (Welch two-sample t-test, $t(31.7) = 0.358$; $p = 0.72$; mean jitter threshold in degrees: Mundurucu 22.86, American 22.49).

References

1. Troje NF (2002) Decomposing biological motion: a framework for analysis and

synthesis of human gait patterns. J Vis 2: 371-387.

2. Field DJ, Hayes A, Hess RF (1993) Contour integrations by the human visual system: evidence for a local “association” field. Vision Res 33: 173-193.
3. Kim J, Doop ML, Blake R, Park S (2005) Impaired visual recognition of biological motion in schizophrenia. Schizophr Res 77: 299-307.
4. Blake R, Turner LM, Smoski MJ, Pozdol SL, Stone WL (2003) Visual recognition of biological motion is impaired in children with autism. Psychol Sci 14: 151-157.
